# Carbene Routes to Cyclopropatetrahedrane

**DOI:** 10.1021/acs.joc.2c02217

**Published:** 2022-11-29

**Authors:** Murray G. Rosenberg, Udo H. Brinker

**Affiliations:** † Institute of Organic Chemistry, University of Vienna, Währinger Strasse 38, 1090 Vienna, Austria; § Department of Chemistry, The State University of New York at Binghamton, P.O. Box 6000, Binghamton, New York 13902-6000, United States

## Abstract

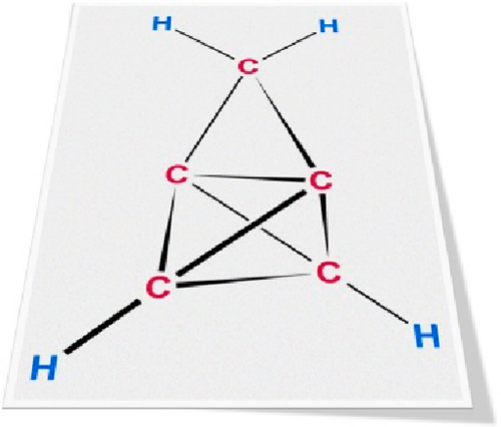

The formation of cyclopropatetrahedrane (tetracyclo[2.1.0.0^1,3^.0^2,4^]pentane) via four different carbene reactions is computed using the (U)CCSD(T)(full)/cc-pVTZ//(U)ωB97X-D/cc-pVTZ + 1.3686(*E*_ZPVE_) theoretical model. Intrinsic reaction coordinate plots confirm that each carbene is directly linked to cyclopropatetrahedrane via a unique cyclopropanation step. Each elementary step is assessed according to the structure and energy of its transition state.

This report assesses four carbene reactions that ostensibly could form cyclopropatetrahedrane (**1**;^1^Figure 1a),^2,3^ a cyclopropane-fused derivative of tetrahedrane (**2**; Figure 1b).^4−7^ To date, **1** and **2** remain hypothetical constructs, although derivatives of **2**(4) as well as pristine **3** and **4** (Figure 1c,d)^8,9^ have been prepared (cf. Table S1 in Supporting Information). Nevertheless, earlier computations suggest that **1** will be kinetically stable because (1) it occupies a deep energy minimum on the C_5_H_4_ hypersurface and (2) none of its 21 vibrational normal modes falls below ν̅ = 443 cm^–1^.^2,3^ Forming **1** will be challenging because (1) its computed strain energy (Δ_strain_*H*° = 157 kcal/mol)^2a^ is phenomenal and (2) the bridging CH_2_–group of **1** establishes a bond that connects two inverted C atoms (i.e., each C atom has four bonds pointing in the same direction;^10−12^Figure 1a; cf. Figures S2 and S3 in Supporting Information). Also, the long C1–C4 bond (*r* = 1.664 Å)^2a^ of **1** is electron-depleted, weak, and prone to breakage when compared with typical aliphatic C–C bonds. Routes to **1** have been proposed, such as via a 2,4-dihalotricyclo[1.1.1.0^1,3^]pentane synthon (**5**; Scheme 1).^4,5^ However, carbene routes to **1** have never been investigated.

**Figure 1:**
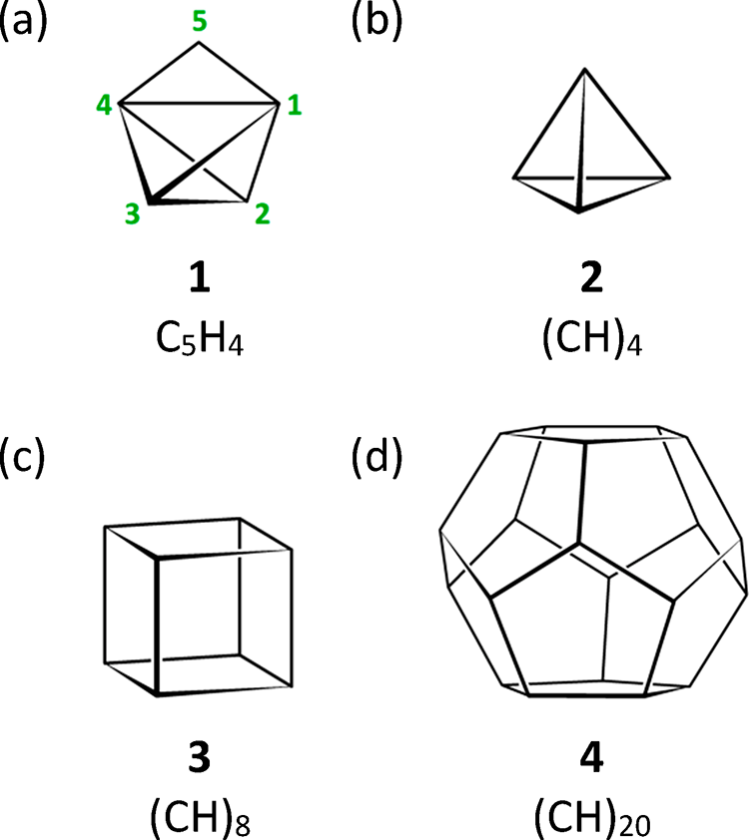
Cyclopropatetrahedrane (**1**) is a cyclopropane-fused derivative of tetrahedrane (**2**), which itself is one of the Platonic-solid-like hydrocarbons that also include cubane (**3**) and dodecahedrane (**4**).

**Scheme 1:**
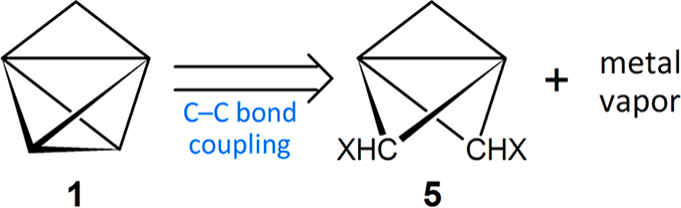
A Proposed Retrosynthesis of Cyclopropatetrahedrane (**1**)

Carbene reaction intermediates are uncharged, electron-deficient, and highly energetic.^13−27^ They are prized for their ability to form a wide variety of cyclopropanes, which can be done in two ways. The divalent C atom (**:C**<) can (1) insert into a homovicinal C–H bond (e.g., carbene **6** → **2**) or (2) add to a C–C double bond (e.g., carbene **7** → **2**) (Scheme 2).^28^ These two signature reactions are useful when building polycycloalkanes. Thus, an examination of carbene routes to highly strained **1** is warranted.

**Scheme 2:**
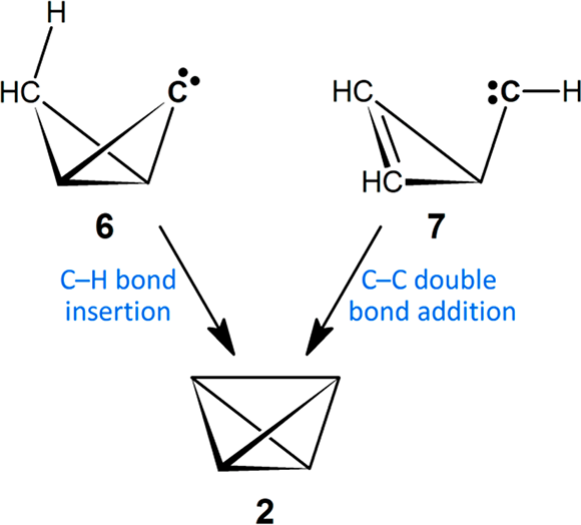
Types of Intramolecular Carbene Cyclopropanations

Four routes to **1** via four different hypothetical carbene reaction intermediates (Scheme 3) were evaluated using the (U)CCSD(T)(full)/cc-pVTZ//(U)ωB97X-D/cc-pVTZ + 1.3686(*E*_ZPVE_) theoretical model (see Computational Methods). Paths a–c depict homovicinal C–H bond insertion reactions within carbenes **8**–**10**, respectively, and path d depicts a C–C double bond addition reaction within carbene **11**. The structures in Scheme 3 are drawn in a uniform manner to emphasize the new bonds being formed (cf. Figure 2): (1) path a, C_**α**_–C_**β**_; (2) path b, C_**γ**_–C_**γ**_; (3) path c, C_**β**_–C_**γ**_; and (4) path d, C_**α**_–C_**β**_ and C_**β**_–C_**β**_. Each elementary step is characterized in terms of its transition state (TS) structure, activation energy (*E*_a_), and net energy change (Δ*E*) (Table 1). Intrinsic reaction coordinate (IRC) plots (Figure 3a–d) and videos (see Supporting Information) are also provided to demonstrate that each carbene is directly linked to **1**.

**Scheme 3:**
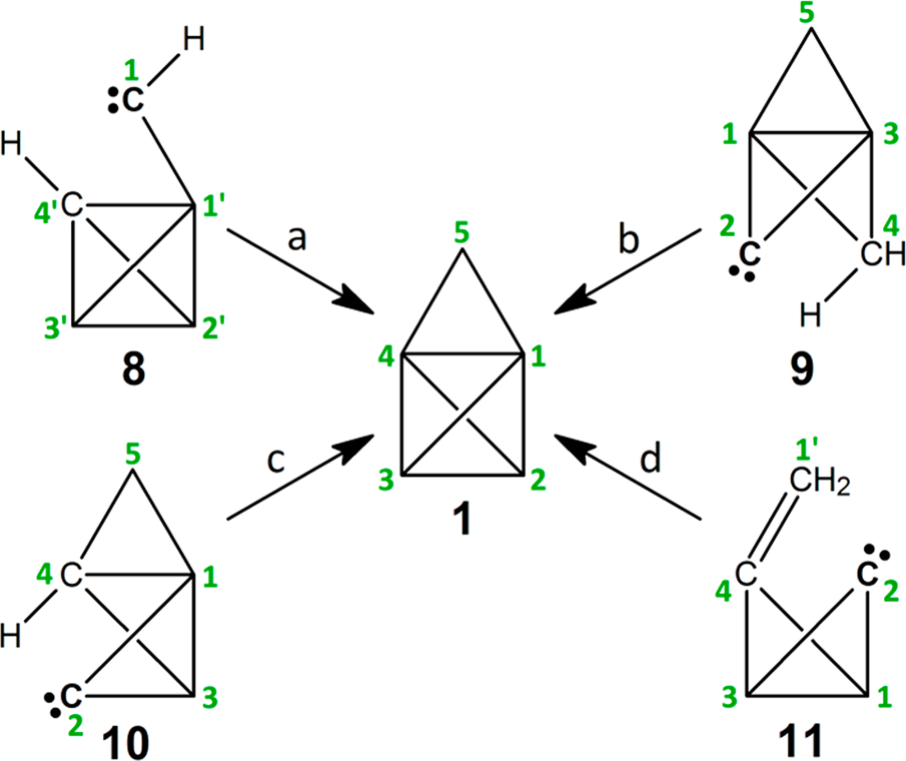
Four Carbene Routes to Cyclopropatetrahedrane (**1**)

**Figure 2:**
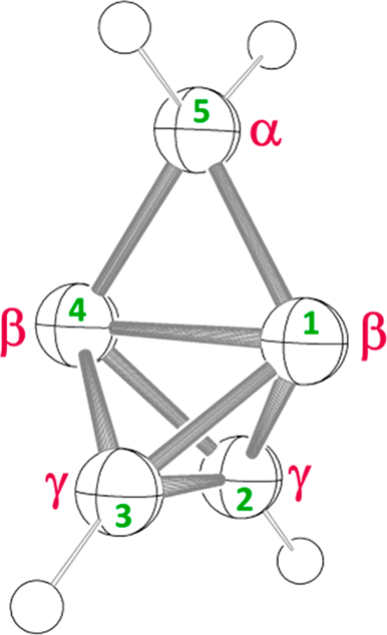
*C*_2*v*_-symmetric cyclopropatetrahedrane (**1**) comprises (a) one 2°-C atom (α), (b) two 4°-C atoms (β), and (c) two 3°-C atoms (γ). (ORTEP structure shows 50% ellipsoids.)

**Table 1:** Computed Data for Carbene Isomerizations to **1**^a^^,^^b^

Carbene	IRC path	ν̅ _TS_ (cm^–1^)	*E*_a_ (kcal/mol)	Δ*E* (kcal/mol)
**8**	a	1164*i*	15.8	–50.2
**9**	b	915*i*	14.2	–24.5
**10**	c	961*i*	3.5	–29.2
**11**	d	329*i*	27.8	10.0

a Cf. Scheme 3.

b CCSD(T)(full)/cc-pVTZ//ωB97X-D/cc-pVTZ + 1.3686(*E*_ZPVE_) theoretical model.

**Figure 3:**
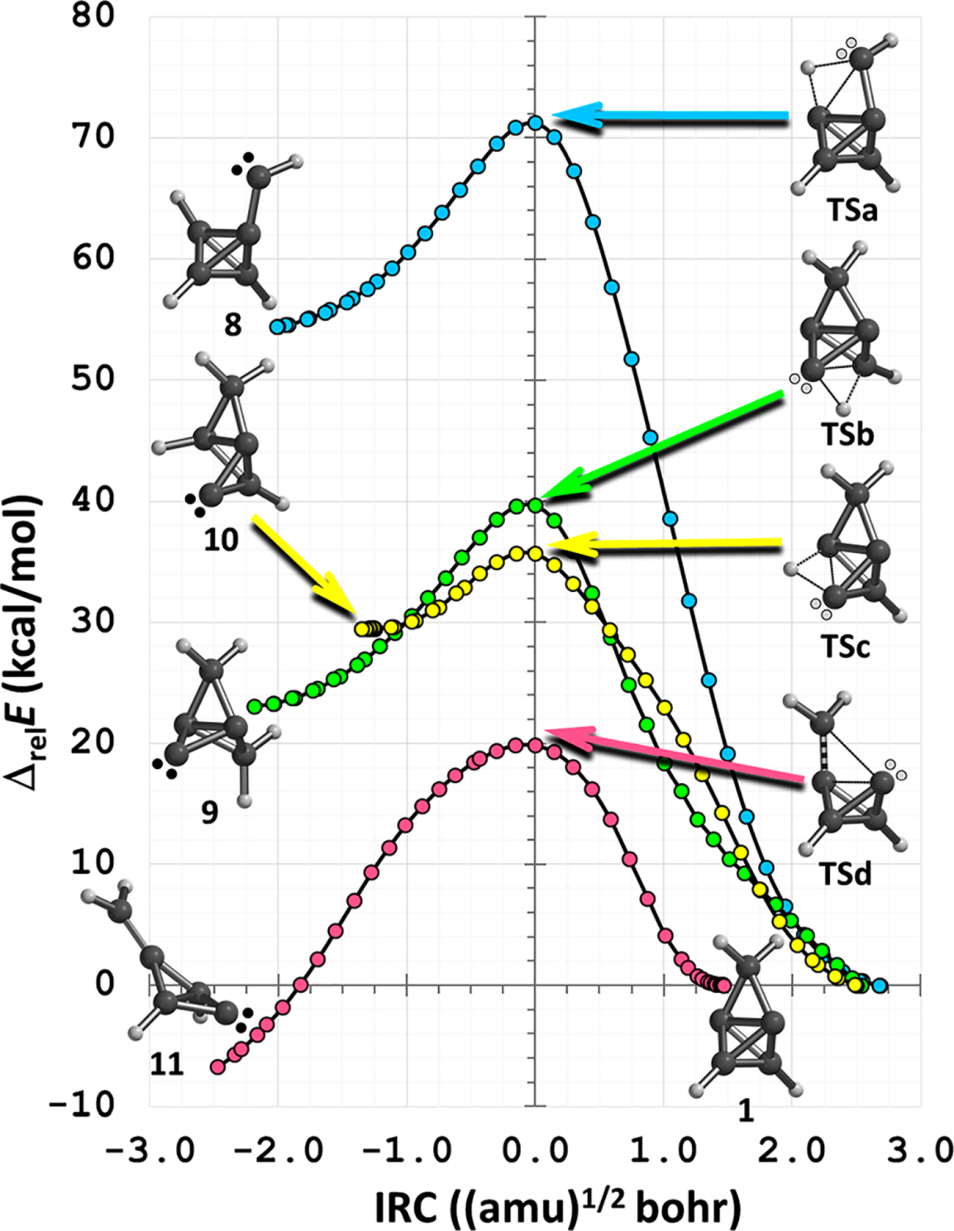
Four IRCs were computed using the CCSD(T)/cc-pVTZ//ωB97X-D/cc-pVTZ theoretical model. Routes (a)–(c) depict homovicinal C–H bond insertion reactions within carbenes **8**–**10**, respectively, while route (d) depicts a C–C double bond addition reaction within carbene **11**.

Path a involves the hypothetical carbene (tetrahedryl)carbene (**8**).^29^ A homovicinal C–H bond insertion reaction via **TSa** was confirmed by its one, and only one, imaginary frequency (Table 1, path a), by animating the corresponding vibration, and by plotting the IRC, which links **8** directly to **1** (i.e., **8** → **TSa** → **1**; Figure 3a (blue)).

An intriguing aspect of the carbene itself was found. Its computed singlet–triplet energy gap (Δ*E*_S–T_)^30^ of −6.7 kcal/mol, corrected for the experimental Δ*E*_S–T_ of CH_2_ (eq 1; see Supporting Information),^31^ indicates that alkylcarbene **8** has a singlet ground state, and decidedly so. Hyperconjugation^32−35^ between the C1′–C4′ “banana” bond and the vacant p orbital of the carbene’s divalent C atom is a contributing factor (cf. Figure S1 in Supporting Information). The :CH-group of the lowest energy conformation of **8** is *bent* 41 deg toward the C1′–C4′ bond in comparison to the ·C·H-group of triplet (tetrahedryl)carbene (i.e., ^3^**8**) (Scheme 3, path a) even though this deformation causes the C1′ atom’s four bonds to point in one direction (i.e., C1′ is an inverted C atom; cf. Figure S2 in Supporting Information). The distorted geometry of **8** may assist the formation of **TSa** since a triangular array comprising the C1, C1′, and C4′ atoms is already established (Figure 3a (blue)). Thus, the high Δ*H*^⧧^ may be more prohibitive than Δ*S*^⧧^ for the homovicinal C–H bond insertion reaction **8** → **1**.
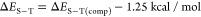
1

Path b involves the hypothetical carbene tricyclo[1.1.1.0^1,3^]pent-2-ylidene (**9**). A homovicinal C–H bond insertion reaction via **TSb** was confirmed by its one, and only one, imaginary frequency (Table 1, path b), by animating the corresponding vibration, and by plotting the IRC, which links **9** directly to **1** (i.e., **9** → **TSb** → **1**; Figure 3b (green)).

Path c involves the hypothetical carbene *trans*-tricyclo[2.1.0.0^1,3^]pent-2-ylidene (**10**). A homovicinal C–H bond insertion reaction via **TSc** was confirmed by its one, and only one, imaginary frequency (Table 1, path c), by animating the corresponding vibration, and by plotting the IRC, which links **10** directly to **1** (i.e., **10** → **TSc** → **1**; Figure 3c (yellow)).

Path d involves the hypothetical carbene 4-methylenebicyclo[1.1.0]but-2-ylidene (**11**). A cycloaddition reaction via **TSd** was confirmed by its one, and only one, imaginary frequency (Table 1, path d), by animating the corresponding vibration, and by plotting the IRC, which links **11** directly to **1** (i.e., **11** → **TSd** → **1**; Figure 3d (red)). However, in contrast to those of the homovicinal C–H bond insertion reactions (Table 1, paths a–c), the net Δ*E* computed for this elementary step is positive. This indicates a thermodynamic preference for a *cycloreversion* of **1** (i.e., **1** → **11**). However, **1** → **11** is computed to have a high Δ*H*^‡^ (18.1 kcal/mol; see Supporting Information). Of course, this enthalpy barrier is not insurmountable even in a frozen Ar matrix (*T* = ca. 10 K) under photolytic conditions.^36^

Computational chemistry was used to assess the viability of forming cyclopropatetrahedrane (**1**) via four different carbene reactions. The hypothesis appears to be valid because a TS was found for each of the elementary steps (i.e., Scheme 3, paths a–d). Furthermore, the respective IRC plots (Figure 3a–d) reveal a direct link between each carbene and **1**. The IRCs and ZPVE-corrected single-point energies show that the homovicinal C–H bond insertion reactions via H atom transfer are exothermic but the C–C double bond addition reaction is endothermic. The formation of **1** via a homovicinal C–H bond insertion within *trans*-tricyclo[2.1.0.0^1,3^]pent-2-ylidene (**10**) requires an *E*_a_ of just 3.5 kcal/mol. The bent posture adopted by the electron-seeking :CH-group of (tetrahedryl)carbene (**8**) is akin to a house plant that is bent toward a sunlit window; each “stalk” bends to obtain what it needs. In contrast, stabilizing hyperconjugation is precluded in triplet (tetrahedryl)carbene (^3^**8**) because of its half-occupied p orbital. Thus, the triplet carbene is strictly *C*_*s*_-symmetric.

## Computational Methods

Quantum chemical calculations were performed on **1**, carbenes **8**–**11**, transition states **TSa**–**TSd**, and intrinsic reaction coordinate (IRC) paths a–d using the Spartan’20 (v. 1.1.4) computer program.^37^ Restricted SCF wave functions of molecular equilibrium geometries and transition states were computed using a (100,434) DFT integration grid, the RSH-GGA functional ωB97X-D,^38^ and Dunning’s cc-pVTZ basis set. Unrestricted SCF wave functions were computed for triplet-state carbenes. Normal-mode vibrational analyses were performed at the level of geometry optimization. The harmonic frequencies were used to obtain temperature-independent zero-point vibrational energy (*E*_ZPVE_)^39^ and temperature-dependent thermal vibrational energy (Δ_vib_*H*) values. Each reaction TS had one, and only one, imaginary frequency, ν̅ _TS_. Its vibration was animated to verify that the motions conformed to the elementary step. An IRC was computed to ensure that the carbene followed a direct route to **1**. Single-point energy (*E*) values were computed using the CCSD(T)(full) coupled-cluster theory method and Dunning’s cc-pVTZ basis set. All *E*_ZPVE_ values were scaled by *z* = 1.3686^40^ before being added to *E* (*T* = 0 K; *p* = 0 atm). Relative energy values (Δ_rel_*E*) are specified with regard to **1** ((Δ_rel_*E* = [0]). Conversion of *E* values to enthalpy (*H*_*T*_) values was done according to eq S1 (see Supporting Information; computational standard state: *T* = 298.15 K; *p* = 1 atm; cf. Table S2). All Δ_vib_*H* values were scaled by *H* = 0.956^40^ before being added to the ZPVE-corrected *E* values. The increase in kinetic energy, due to translations (3(1/2)R*T*) and rotations (3(1/2)R*T*), for each nonlinear molecule was then added. Finally, *RT* (i.e., “*pV* work” needed to expand 1 mol of ideal gas to *V* = 24.465 L at *T* = 298.15 K and *p* = 1 atm) was added to obtain *H*_T_ (eq S1).

## Data Availability

The data underlying this study are available in the published article and its online Supporting Information.

## Supporting Information Available

Computational methods, Cartesian coordinates, ORTEP structures, energies/enthalpies, and geometric proof of inverted C atoms (PDF)
Energy results (XLSX)
IRC data (XLSX)
Inverted carbon atom data (XLSX)
IRC data and video (wB97X-D_cc-pVTZ.IRC_path a_(Tricyclo[1_1_0_0(2,4)but-1-yl)carbene_(8)) MOV)
IRC data and video (wB97X-D_cc-pVTZ.IRC_path b_Tricyclo[1_1_1_0(1,3)]pent-2-ylidene_(9)) (MOV)
IRC data and video (wB97X-D_cc-pVTZ.IRC_path c_Tricyclo[2_1_0_0(1,3)]pent-2-ylidene_(10)) (MOV)
IRC data and video (wB97X-D_cc-pVTZ.IRC_path d_4-Methylenebicyclo[1_1_0]but-2-ylidene_(11)) (MOV)

## Supplementary Material

jo2c02217_si_004.xlsx

jo2c02217_si_005.mov

jo2c02217_si_006.mov

jo2c02217_si_007.mov

jo2c02217_si_008.mov
