# Supplementary material for: Carbene Routes to Cyclopropatetrahedrane
Source: J Org Chem. 2022 Nov 29;87(24):16902–6. doi: 10.1021/acs.joc.2c02217 (PMC9764353; doi:10.1021/acs.joc.2c02217)
Supplement: Supplementary file 1 — jo2c02217_si_001.pdf [file jo2c02217_si_001.pdf]

Carbene Routes to Cyclopropatetrahedrane

Murray G. Rosenberg[§] and Udo H. Brinker[*,†,§]
[†]Institute of Organic Chemistry, University of Vienna, Währinger Strasse 38, 1090 Vienna, Austria
[§]Department of Chemistry, The State University of New York at Binghamton, P. O. Box 6000, Binghamton, NY, 13902-6000, United States
[*]E-mail: udo.brinker@univie.ac.at; ubrinker@binghamton.edu

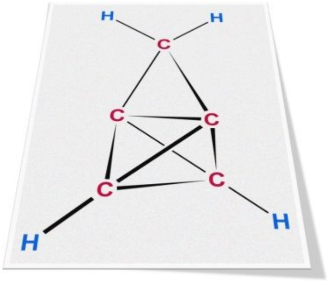

SUPPORTING INFORMATION

Platonic Solids S2
Computational Modeling S3
Molecular Energies and 3-D Cartesian Coordinates S5
Molecular Orbitals of Select Molecules S12
Geometric Analysis of Inverted Carbon Atoms S13

PLATONIC SOLIDS

Tetrahedrane (**2**), hexahedrane (**3**; cubane), and dodecahedrane (**4**) are fused polycyclic hydrocarbons (Table S1).[S1] Their cage-like C-atom frameworks correspond to a regular (1) tetrahedron, (2) hexahedron (i.e., cube), and (3) dodecahedron, which are three of the five regular polyhedra that have been considered sacred since the time of the ancient Greeks. The syntheses of **3** and **4** are a remarkable achievement despite the structures' high symmetries and total strain energies (E_s)s.[S2] However, **2** has never been prepared although polysubstituted derivatives are known.[S3] The six curved C−C "banana" bonds of **2** have high p orbital character and bow outside the C−C internuclear axes by 21 deg to relieve strain. The E_s per C−C bond value for **2** exceeds that of **3** and far exceeds that of **4**.

Table S1. Comparison of Platonic-Solid-Like Hydrocarbons[a]

$(CH)_v$	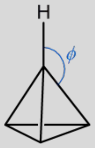	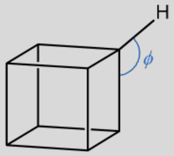	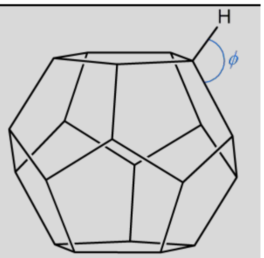
	**2**	**3**	**4**
symmetry	T_d	O_h	I_h
faces (f)	4	6	12
C−H units (v)	4	8	20
C−C bonds (e)	6	12	30
SODAR[b]	3	5	11
ϕ (deg)	144.74	125.26	110.91
E_s (kcal/mol):			
(a) total[c]	136.18	159.99	60.45
(b) per C−C bond	22.70	13.33	2.02

[a] Euler's formula: $f + n - 2 = e$. [b] Sum of double bonds and rings (SODAR) for $(CH)_v$ is $[(v/2) + 1]$ and is equivalent to $(f - 1)$. [c] cf. Ref. S2.

COMPUTATIONAL MODELING

Computational Methods. Quantum chemical calculations were performed on **1**, carbenes **8**−**11**, transition states **TSa**−**TSd**, and intrinsic reaction coordinate (IRC) paths a−d using the *Spartan'20* (v. 1.1.4) computer program.[S4] Restricted SCF wave functions of molecular equilibrium geometries and transition states were computed using a (100,434) DFT integration grid, the RSH-GGA functional ωB97X-D,[S5] and Dunning's cc-pVTZ basis set. Unrestricted SCF wave functions were computed for triplet-state carbenes. Normal-mode vibrational analyses were performed at the level of geometry optimization. The harmonic frequencies were used to obtain temperature-independent zero-point vibrational energy (E_{ZPVE})[S6] and temperature-dependent thermal vibrational energy ($\Delta_{vib}H$) values. Each reaction TS had one, and only one, imaginary frequency $\bar{\nu}_{TS}$. Its vibration was animated to verify that the motions conformed to the elementary step. An IRC was computed to ensure that the carbene followed a direct route to **1**. Single-point energy (E) values were computed using the CCSD(T)(full) coupled-cluster theory method and Dunning's cc-pVTZ basis set. All E_{ZPVE} values were scaled by Z = 1.3686[S7] before being added to E (T = 0 K; p = 0 atm). Relative energy values ($\Delta_{rel}E$)s are specified with regard to **1** (($\Delta_{rel}E$ = [0]). Conversion of E values to enthalpy (H_T) values was done according to eq S1 (computational standard-state: T = 298.15 K; p = 1 atm; cf. Table S2). All $\Delta_{vib}H$ values were scaled by H = 0.956[S7] before being added to the ZPVE-corrected E values. The increase in kinetic energy, due to translations (3(½)RT) and rotations (3(½)RT), for each nonlinear molecule was then added. Finally, RT (*i.e.*, "*pV* work" needed to expand one mole of ideal gas to V = 24.465 L at T = 298.15 K and p = 1 atm) was added to obtain H_T (eq S1). The experimental singlet−triplet energy gap (ΔE_{S-T}) of CH_2 (eq S2)[S8] was used to compute the corrected ΔE_{S-T} of carbene **8** (eq S3).

Table S2. Values Used in Computations with Equation S1

Parameter		Value
V	=	nRT/p
	=	24.465 L
p	=	1 atm
n	=	1 mol
R	=	1.9872×10^{-3} (kcal/mol)/K
	=	8.2057×10^{-2} (L·atm/mol)/K
T	=	298.15 K
	=	25 °C
RT	=	0.592 kcal/mol
3(½)RT	=	0.889 kcal/mol
1 hartree	=	627.5095 kcal/mol

$$H_T = [E + \text{Z}(E_{\text{ZPVE}})] + \text{H}(H_{\text{vib}}) + (3(½)\text{R}T)_{\text{translational}} + (3(½)\text{R}T)_{\text{rotational}} + (\text{R}T)_{\text{ideal gas}} \quad \text{(S1)}$$

$$\Delta E_{\text{S}-\text{T}} = \Delta E_{\text{S}-\text{T(comp)}} - [\Delta E_{\text{S}-\text{T(comp)}} - \Delta E_{\text{S}-\text{T(exp't)}}]_{\text{CH}_2} \quad \text{(S2)}$$

$$\Delta E_{\text{S}-\text{T}} = \Delta E_{\text{S}-\text{T(comp)}} - 1.247 \text{ kcal/mol} \quad \text{(S3)}$$

[S9]

MOLECULAR ENERGIES AND 3-D CARTESIAN COORDINATES

All ORTEP structures are shown as 50% ellipsoids.

Methylene ($^1CH_2$);
CCSD(T)(full)/cc-pVTZ//ωB97X-D/cc-pVTZ + 1.3686(E_{ZPVE}):

E + z(E_{ZPVE}): −24505.30 kcal/mol

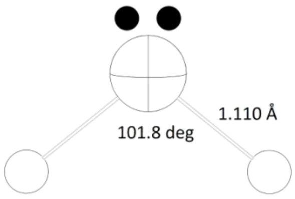

<u>*T* = 298.15 K</u>

H_T: −24502.92 kcal/mol

Atom	*x*	*y*	*z*
C	0.0000000	0.0000000	0.1820529
H	0.8612946	0.0000000	-0.5174490
H	-0.8612946	0.0000000	-0.5174490

Methanediyl ($^3CH_2$);
UCCSD(T)(full)/cc-pVTZ//UωB97X-D/cc-pVTZ + 1.3686(E_{ZPVE}):

E + z(E_{ZPVE}): −24502.92 kcal/mol

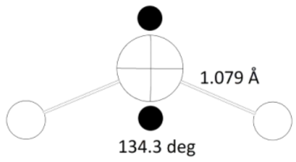

<u>*T* = 298.15 K</u>

H_T: −24513.37 kcal/mol

Atom	*x*	*y*	*z*
C	0.0000000	0.0000000	0.1036352
H	0.9943111	0.0000000	-0.3150600
H	-0.9943111	0.0000000	-0.3150600

Cyclopropatetrahedrane (**1**; tetracyclo[2.1.0.0^{1,3}.0^{2,4}]pentane);
CCSD(T)(full)/cc-pVTZ//ωB97X-D/cc-pVTZ + 1.3686(E_{ZPVE}):

E + z(E_{ZPVE}): −120693.23 kcal/mol

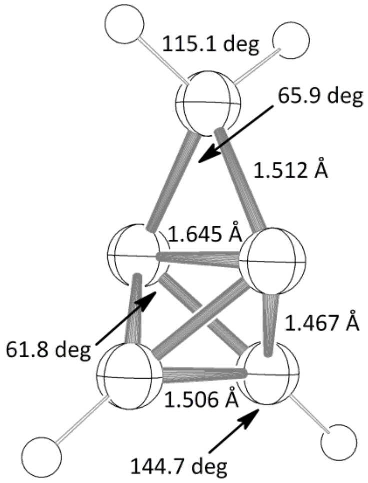

T = 298.15 K

H_T: −120690.22 kcal/mol

Atom	*x*	*y*	*z*
C	0.0000000	0.0000000	−1.3639010
C	0.0000000	0.8222895	−0.0944911
C	0.0000000	−0.8222895	−0.0944911
C	0.7530674	0.0000000	0.8583930
C	−0.7530674	0.0000000	0.8583930
H	1.6266535	0.0000000	1.4771491
H	−1.6266535	0.0000000	1.4771491
H	0.9145992	0.0000000	−1.9456671
H	−0.9145992	0.0000000	−1.9456671

(Tetrahedryl)carbene (**8**; (tricyclo[1.1.0.0^{2,4}]but-1-yl)methylene);
CCSD(T)(full)/cc-pVTZ//ωB97X-D/cc-pVTZ + 1.3686(E_{ZPVE}):

E + z(E_{ZPVE}): −120643.03 kcal/mol

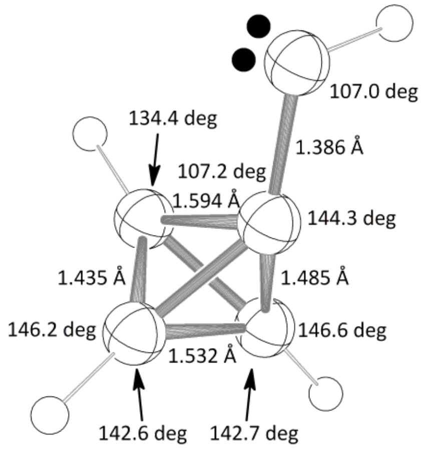

T = 298.15 K

H_T: −120639.55 kcal/mol

Atom	*x*	*y*	*z*
C	0.4406085	0.0534540	0.4349542
C	-0.7894082	-0.7700605	0.3130382
C	-0.8286348	0.7593716	0.2357073
C	-0.4482145	-0.0546304	-0.8835848
C	1.7616002	0.1706280	0.0309249
H	-1.3354576	-1.6191480	0.6690817
H	-1.4240687	1.6089756	0.4977220
H	-0.2328387	-0.0911725	-1.9323936
H	2.1754413	-0.8521786	-0.0208441

(Tetrahedryl)carbene triplet (^{3}**8**; (tricyclo[1.1.0.0^{2,4}]but-1-yl)methanediyl);
UCCSD(T)(full)/cc-pVTZ//UωB97X-D/cc-pVTZ + 1.3686(E_{ZPVE}):

E + z(E_{ZPVE}): −120637.55 kcal/mol

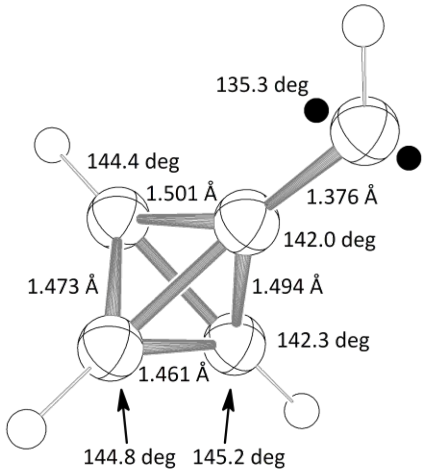

T = 298.15 K

H_T: −120633.81 kcal/mol

Atom	*x*	*y*	*z*
C	0.7644593	-0.4614847	0.7362688
C	0.7644593	-0.4614847	-0.7362688
C	0.7829180	0.8017868	0.0000000
C	-0.4659120	-0.0176167	0.0000000
C	-1.8368338	0.0951632	0.0000000
H	1.1310111	1.8123758	0.0000000
H	-2.6616046	-0.5976657	0.0000000
H	1.1198584	-0.9706518	1.6063754
H	1.1198584	-0.9706518	-1.6063754

Tricyclo[1.1.1.0^{1,3}]pent-2-ylidene (**9**);
CCSD(T)(full)/cc-pVTZ//ωB97X-D/cc-pVTZ + 1.3686(E_{ZPVE}):

E + z(E_{ZPVE}): −120668.71 kcal/mol

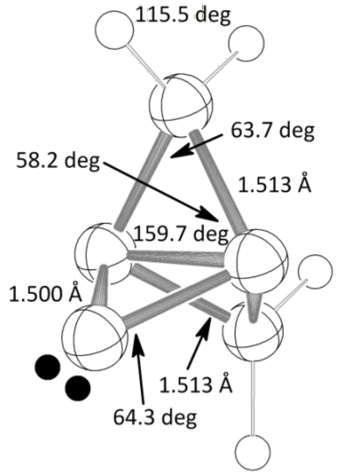

T = 298.15 K

H_T: −120665.41 kcal/mol

Atom	*x*	*y*	*z*
C	0.0000000	0.0000000	-1.3697075
C	1.1249653	0.0000000	0.5209824
C	-1.1249653	0.0000000	0.5209824
C	0.0000000	0.7983562	-0.1004006
C	0.0000000	-0.7983562	-0.1004006
H	1.1642895	0.0000000	1.6035277
H	-1.1642895	0.0000000	1.6035277
H	2.0836611	0.0000000	0.0194302
H	-2.0836611	0.0000000	0.0194302

trans-Tricyclo[2.1.0.0^{1,3}]pent-2-ylidene (**10**);
CCSD(T)(full)/cc-pVTZ//ωB97X-D/cc-pVTZ + 1.3686(E_{ZPVE}):

E + z(E_{ZPVE}): −120664.05 kcal/mol

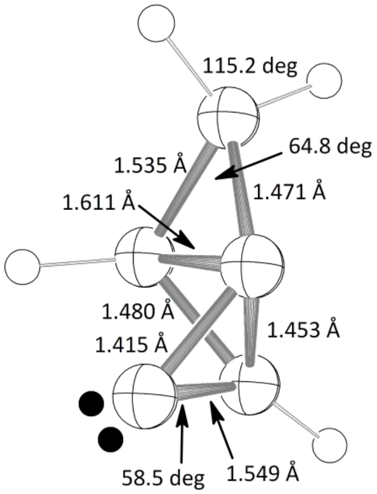

T = 298.15 K

H_T: −120660.90 kcal/mol

Atom	*x*	*y*	*z*
C	-0.0081795	-0.4345073	0.5950044
C	0.8744954	0.6582343	0.2245362
C	1.1400068	-0.8247856	-0.1336219
C	-0.2158522	0.4282309	-0.7497125
C	-1.3596092	-0.0553002	0.1534798
H	1.3233488	1.5021016	0.7145638
H	-0.0419469	-0.0906870	-1.6957574
H	-2.0194039	-0.8153141	-0.2542960
H	-1.8474828	0.7116628	0.7430040

4-methylenebicyclo[1.1.0]but-2-ylidene (**11**);
CCSD(T)(full)/cc-pVTZ//ωB97X-D/cc-pVTZ + 1.3686(E_{ZPVE}):

E + z(E_{ZPVE}): −120703.26 kcal/mol

<u>*T* = 298.15 K</u>

H_T: −120699.94 kcal/mol

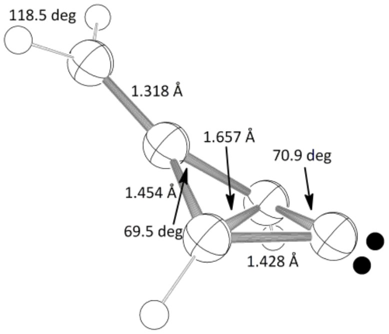

Atom	*x*	*y*	*z*
C	0.6417445	0.1610869	0.8283118
C	0.6417445	0.1610869	-0.8283118
C	1.6208845	-0.4673074	0.0000000
C	-0.5478140	0.0504135	0.0000000
C	-1.8565107	-0.1059036	0.0000000
H	0.8305334	0.8519009	1.6415358
H	0.8305334	0.8519009	-1.6415358
H	-2.4032176	-0.1820810	0.9276798
H	-2.4032176	-0.1820810	-0.9276798

TSa;
CCSD(T)(full)/cc-pVTZ//ωB97X-D/cc-pVTZ + 1.3686(E_{ZPVE}):

E + z(E_{ZPVE}): −120627.19 kcal/mol

<u>*T* = 298.15 K</u>

H_T: −120623.84 kcal/mol

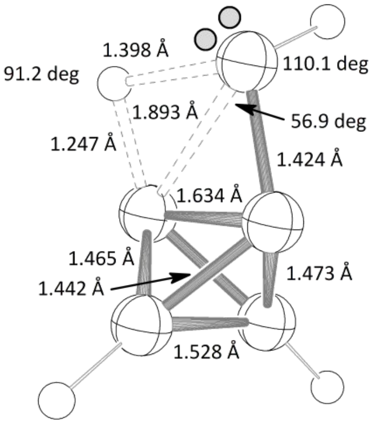

Atom	*x*	*y*	*z*
C	-0.2272812	0.0206392	0.7878857
C	0.8303660	0.7593271	0.0778881
C	0.8690167	-0.7568780	0.2652301
C	0.0844535	-0.1404997	-0.8077560
C	-1.5153744	-0.1410754	0.2032869
H	1.4372237	1.6433148	0.0843197
H	1.5204804	-1.5928724	0.4202580
H	-1.0428854	-0.6298454	-1.0178656
H	-1.9559995	0.8378898	-0.0132471

TSb;
CCSD(T)(full)/cc-pVTZ//ωB97X-D/cc-pVTZ + 1.3686(E_{ZPVE}):

E + z(E_{ZPVE}): −120654.53 kcal/mol

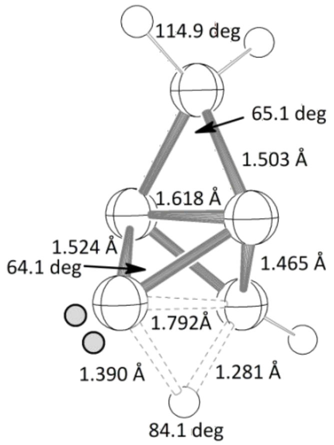

<u>T = 298.15 K</u>

H_T: −120651.29 kcal/mol

Atom	x	y	z
C	-0.2098599	0.8088502	0.0114610
C	-0.2098599	-0.8088502	0.0114610
C	-1.2373062	0.0000000	-0.7721676
C	-0.0573475	0.0000000	1.2688401
C	0.5456639	0.0000000	-0.9478803
H	1.5147359	0.0000000	-1.4153330
H	-0.3621737	0.0000000	-1.8522700
H	0.9152113	0.0000000	1.7444079
H	-0.8990640	0.0000000	1.9514810

TSc;
CCSD(T)(full)/cc-pVTZ//ωB97X-D/cc-pVTZ + 1.3686(E_{ZPVE}):

E + z(E_{ZPVE}): −120660.59 kcal/mol

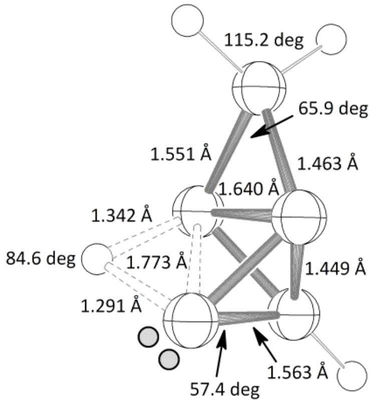

<u>T = 298.15 K</u>

H_T: −120657.34 kcal/mol

Atom	x	y	z
C	-0.0722746	-0.1214417	0.7605083
C	0.9097646	0.6776172	0.0553228
C	0.9431212	-0.8846241	0.0713048
C	-0.1190331	0.1966518	-0.8479002
C	-1.3549160	0.0449137	0.0761435
H	1.5715340	1.5120681	0.1769667
H	0.3800498	-1.0266014	-1.0821693
H	-1.9119569	0.9582075	0.2520532
H	-1.9603343	-0.8389315	-0.0975726

TSd;
CCSD(T)(full)/cc-pVTZ//ωB97X-D/cc-pVTZ + 1.3686(E_{ZPVE}):

E + z(E_{ZPVE}): −120675.45 kcal/mol

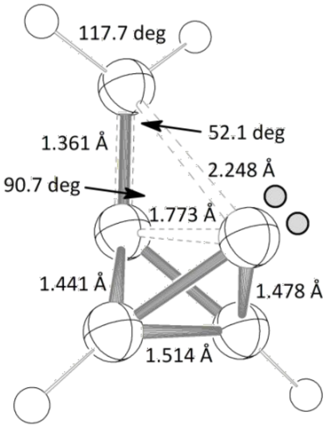

T = 298.15 K

H_T: −120672.11 kcal/mol

Atom	*x*	*y*	*z*
C	0.1495165	0.7571575	-0.9634904
C	0.1495165	-0.7571575	-0.9634904
C	0.5058738	0.0000000	0.2100193
C	-0.1057233	0.0000000	1.4264178
C	-1.0685679	0.0000000	-0.6051027
H	0.4378255	1.6270523	-1.5188101
H	0.4378255	-1.6270523	-1.5188101
H	-0.2531333	0.9266041	1.9666334
H	-0.2531333	-0.9266041	1.9666334

Molecular Orbitals of Select Molecules

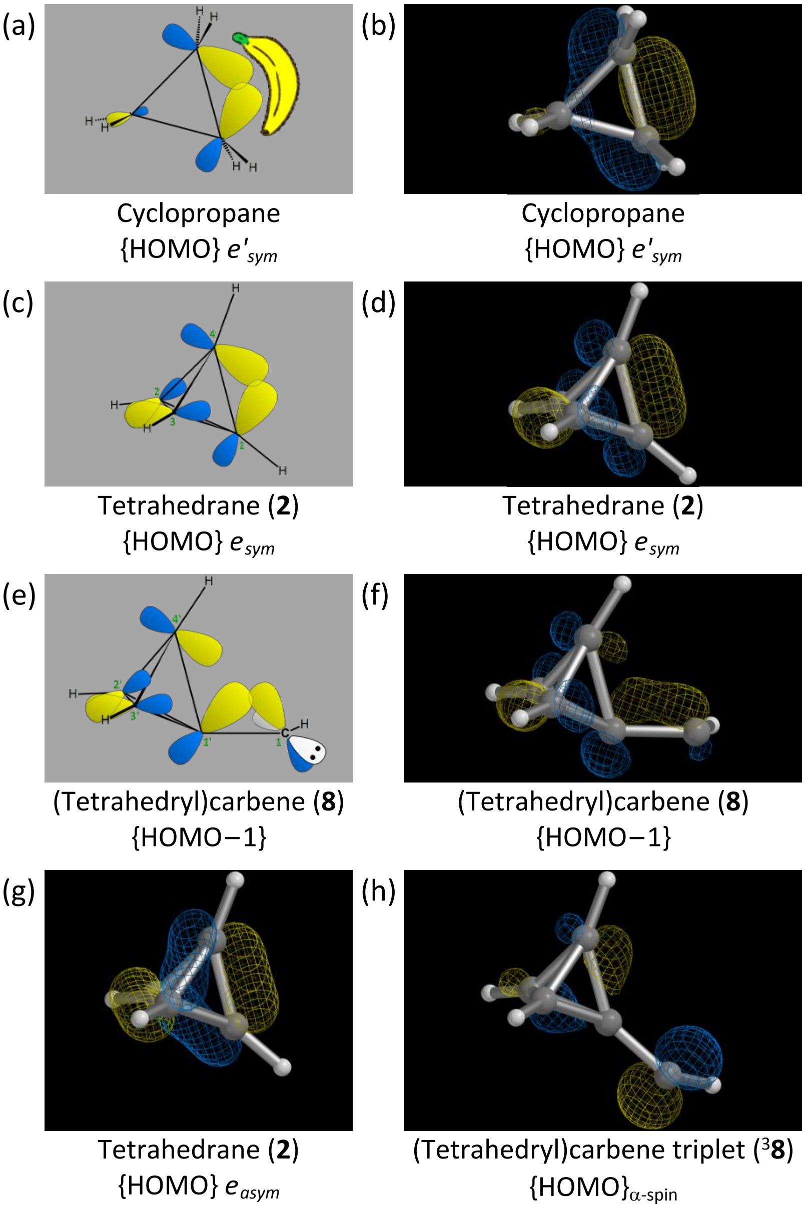

Figure S1. Molecular orbitals (MO)s from select molecules are displayed above. (a, b) Cyclopropane has a high-lying C−C "banana" bond. The outwardly curved σ/π bond is a hybrid between a C−C σ bond and a C−C π bond. (c, d) Tetrahedrane (**2**) also has high-lying C−C "banana" bonds. (e, f) In (tetrahedryl)carbene (**8**), the vacant p orbital of the **:**CH-group attracts electron density from the vicinal C−C "banana" bond. This elongates and weakens the C1'−C4' bond of the tetrahedryl group. (g, h) The **:**CH-group's p orbital is *not* vacant in triplet (tetrahedryl)carbene (^{3}**8**). Its **:**CH-group does *not* bend toward the C1'−C4' bond because a nodal plane exists between the tetrahedryl group and the singly occupied p orbital. All structures and MOs were computed using the (U)ωB97X-D/cc-pVTZ //(U)ωB97X-D/cc-pVTZ theoretical model. All computer-generated MOs are shown with an isosurface value of 0.110 to emphasize the cores of the electron-clouds.

Geometric Analysis of Inverted Carbon Atoms

Below is a straightforward procedure for determining whether the four bonds emanating from a tetracoordinate carbon atom are monohemispherical. Carbene **8** is used as an example (see the original spreadsheet file for the detailed formulas).

Example 1:

(1) Map von Baeyer atom labels to those of computed Cartesian coordinates: C1' → C1, etc. Translate atom of interest (e.g., C1) to the origin (0, 0, 0) by subtracting its (x, y, z) coordinates from all other atoms.

(2) Calculate cross product **u** × **v** (in that order) to find **t**, which is perpendicular to both **u** and **v**.

(3) Compute ϕ_{st} and ϕ_{rt} from the dot products **s** • **t** and **r** • **t**, respectively.

(4) Tetracoordinate C1 is an inverted carbon atom if $\pi/2 < \phi_{st} < 3\pi/2$ and $\pi/2 < \phi_{rt} < 3\pi/2$.

(Tetrahedryl)carbene (**8**; (tricyclo[1.1.0.0^{2,4}]but-1-yl)methylene);
ωB97X-D/cc-pVTZ//ωB97X-D/cc-pVTZ:

$Atom_{von\ Baeyer}$		$Atom_{Cartesian}$	*x*	*y*	*z*
**C1'**	→	C1	0.4406085	0.0534540	0.4349542
C2'	→	C2	-0.7894082	-0.7700605	0.3130382
C3'	→	C3	-0.8286348	0.7593716	0.2357073
C4'	→	C4	-0.4482145	-0.0546304	-0.8835848
C1	→	C5	1.7616002	0.1706280	0.0309249
H2'	→	H2	-1.3354576	-1.6191480	0.6690817
H3'	→	H3	-1.4240687	1.6089756	0.4977220
H4'	→	H5	-0.2328387	-0.0911725	-1.9323936
H1	→	H5'	2.1754413	-0.8521786	-0.0208441

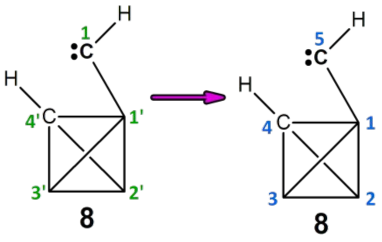

(Tetrahedryl)carbene (**8**);
ωB97X-D/cc-pVTZ

Atom	*x*	*y*	*z*
C1	0.4406085	0.0534540	0.4349542
C2	−0.7894082	−0.7700605	0.3130382
C3	−0.8286348	0.7593716	0.2357073
C4	−0.4482145	−0.0546304	−0.8835848
C5	1.7616002	0.1706280	0.0309249
H2	−1.3354576	−1.6191480	0.6690817
H3	−1.4240687	1.6089756	0.4977220
H5	−0.2328387	−0.0911725	−1.9323936
H5'	2.1754413	−0.8521786	−0.0208441

Step 1

Atom	*x*	*y*	*z*	vector	bond length check<br>norm (Å)
**C1**	**0.0000000**	**0.0000000**	**0.0000000**	—	—
C2	−1.2300167	−0.8235145	−0.1219160	**v**	1.485 Å
C3	−1.2692433	0.7059176	−0.1992469	**u**	1.466 Å
C4	−0.8888230	−0.1080844	−1.3185390	**r**	1.594 Å
C5	1.3209917	0.1171740	−0.4040293	**s**	1.386 Å
H2	−1.7760661	−1.6726020	0.2341275	—	—
H3	−1.8646772	1.5555216	0.0627678	—	—
H5	−0.6734472	−0.1446265	−2.3673478	—	—
H5'	1.7348328	−0.9056326	−0.4557983	—	—

Step 2

Vector	*x*	*y*	*z*	norm (Å)	perpendicularity check<br>dot product	
**u**	−1.2692433	0.7059176	−0.1992469	1.466	0.0000000	**u • t**
**v**	−1.2300167	−0.8235145	−0.1219160	1.485	0.0000000	**v • t**
**t = u × v**	**−0.2501454**	**0.0903359**	**1.9135307**	**1.932**	—	

Step 3

Vector	*x*	*y*	*z*	norm (Å)	dot product	
**s**	1.3209917	0.1171740	−0.4040293	1.386	−0.4080803	**s • t**
**r**	−0.8888230	−0.1080844	−1.3185390	1.594	−0.7503740	**r • t**
					−0.2960112	**s • r**

Step 4

Angle	deg	90 < ϕ < 270	inverted C atom			
ϕ_{st}	114.1	TRUE	—			
ϕ_{rt}	138.6	TRUE	—			
ϕ_{sr}	107.2	—	**TRUE**	by	24.1	deg
sum	359.9					

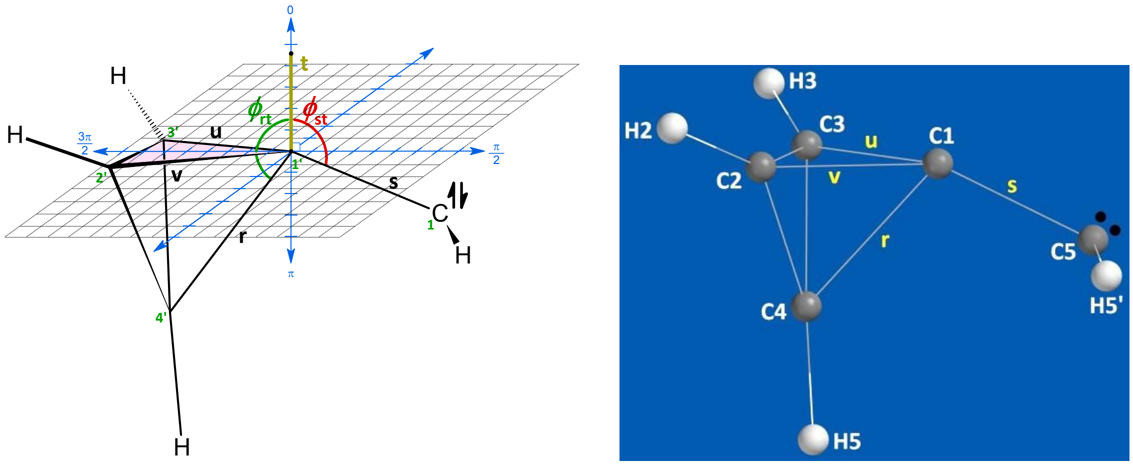

Figure S2. The C1' atom of (tetrahedryl)carbene (**8**) is an inverted carbon atom.

Example 2:

(1) Map von Baeyer atom labels to those of computed Cartesian coordinates: C4' → C4, etc. Translate atom of interest (e.g., C4) to the origin (0, 0, 0) by subtracting its (x, y, z) coordinates from all other atoms.

(2) Calculate cross product **u** × **v** (in that order) to find **t**, which is perpendicular to both **u** and **v**.

(3) Compute ϕ_{st} and ϕ_{rt} from the dot products **s** • **t** and **r** • **t**, respectively.

(4) Tetracoordinate C4 is an inverted carbon atom if $\pi/2 < \phi_{st} < 3\pi/2$ and $\pi/2 < \phi_{rt} < 3\pi/2$.

(Tetrahedryl)carbene (**8**; (tricyclo[1.1.0.0^{2,4}]but-1-yl)methylene);
ωB97X-D/cc-pVTZ//ωB97X-D/cc-pVTZ:

$Atom_{von\ Baeyer}$		$Atom_{Cartesian}$	*x*	*y*	*z*
C1'	→	C1	0.4406085	0.0534540	0.4349542
C2'	→	C2	-0.7894082	-0.7700605	0.3130382
C3'	→	C3	-0.8286348	0.7593716	0.2357073
**C4'**	→	C4	-0.4482145	-0.0546304	-0.8835848
C1	→	C5	1.7616002	0.1706280	0.0309249
H2'	→	H2	-1.3354576	-1.6191480	0.6690817
H3'	→	H3	-1.4240687	1.6089756	0.4977220
H4'	→	H5	-0.2328387	-0.0911725	-1.9323936
H1	→	H5'	2.1754413	-0.8521786	-0.0208441

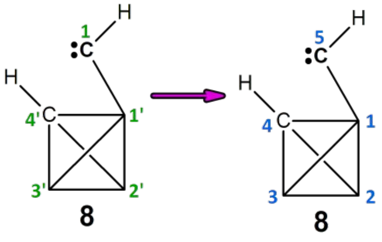

(Tetrahedryl)carbene (**8**);
ωB97X-D/cc-pVTZ

Atom	*x*	*y*	*z*
C1	0.4406085	0.0534540	0.4349542
C2	-0.7894082	-0.7700605	0.3130382
C3	-0.8286348	0.7593716	0.2357073
C4	-0.4482145	-0.0546304	-0.8835848
C5	1.7616002	0.1706280	0.0309249
H2	-1.3354576	-1.6191480	0.6690817
H3	-1.4240687	1.6089756	0.4977220
H5	-0.2328387	-0.0911725	-1.9323936
H5'	2.1754413	-0.8521786	-0.0208441

Step 1 — bond length check

Atom	*x*	*y*	*z*	vector	norm (Å)
C1	0.8888230	0.1080844	1.3185390	r	1.594 Å
C2	-0.3411937	-0.7154301	1.1966230	u	1.435 Å
C3	-0.3804203	0.8140020	1.1192921	v	1.435 Å
**C4**	**0.0000000**	**0.0000000**	**0.0000000**	—	—
C5	2.2098147	0.2252584	0.9145097	—	—
H2	-0.8872431	-1.5645176	1.5526665	—	—
H3	-0.9758542	1.6636060	1.3813068	—	—
H5	0.2153758	-0.0365421	-1.0488088	s	1.071 Å
H5'	2.6236558	-0.7975482	0.8627407	—	—

Step 2 — perpendicularity check

Vector	*x*	*y*	*z*	norm (Å)	dot product	
u	-0.3411937	-0.7154301	1.1966230	1.435	0.0000000	u • t
v	-0.3804203	0.8140020	1.1192921	1.435	0.0000000	v • t
t = u × v	**-1.7748288**	**-0.0733243**	**-0.5498965**	**1.860**	—	

Step 3

Vector	*x*	*y*	*z*	norm (Å)	dot product	
s	0.2153758	-0.0365421	-1.0488088	1.071	0.0989699	s • t
r	0.8888230	0.1080844	1.3185390	1.594	-0.7795955	r • t
					-0.7001050	s • r

Step 4

Angle	deg	90 < ϕ < 270	inverted C atom		
ϕ_{st}	84.3	FALSE	—		
ϕ_{rt}	141.2	TRUE	—		
ϕ_{sr}	134.4	—	FALSE	by	5.7 deg
sum	360.0				

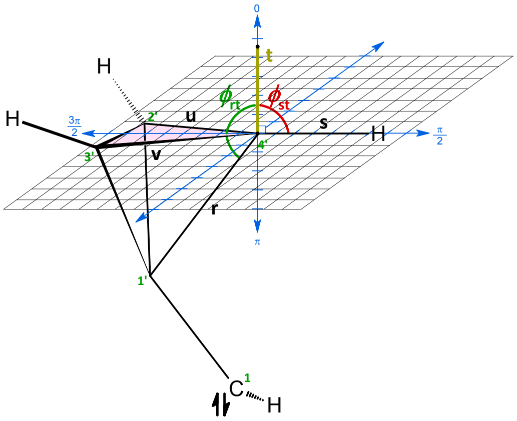

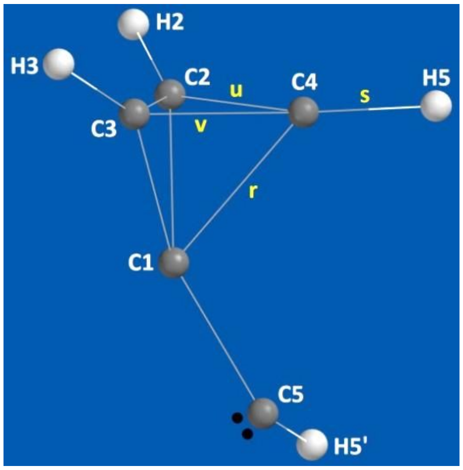

Figure S3. The C4' atom of (tetrahedryl)carbene (**8**) is *not* an inverted carbon atom.

REFERENCES

(S1) Hoffmann, R. How Should Chemists Think? *Sci. Am.* **1993,** *268* (2), 66−73.

(S2) Karton, A.; Schreiner, P. R.; Martin, J. M. L. Heats of Formation of Platonic Hydrocarbon Cages by Means of High-Level Thermochemical Procedures. *J. Comput. Chem.* **2016,** *37,* 49−58.

(S3) (a) Maier, G. Tetrahedrane and Cyclobutadiene. *Angew. Chem., Int. Ed. Engl.* **1988,** *27,* 309−332. (b) Balci, M.; McKee, M. L.; Schleyer, P. v. R. Theoretical Study of Tetramethyl- and Tetra-*tert*-butyl-Substituted Cyclobutadiene and Tetrahedrane. *J. Phys. Chem. A* **2000,** *104,* 1246−1255.

(S4) *Spartan'20,* version 1.1.4; Wavefunction Inc.: Irvine, CA, 2022.

(S5) Chai, J.-D.; Head-Gordon, M. Long-Range Corrected Hybrid Density Functionals with Damped Atom–Atom Dispersion Corrections. *Phys. Chem. Chem. Phys.* **2008,** *10,* 6615−6620.

(S6) Csonka, G. I.; Ruzsinszky, A.; Perdew, J. P. Estimation, Computation, and Experimental Correction of Molecular Zero-Point Vibrational Energies. *J. Phys. Chem. A* **2005,** *109,* 6779−6789.

(S7) Computational Chemistry Comparison and Benchmark DataBase. National Institute of Standards and Technology. http://cccbdb.nist.gov/introx.asp (accessed Aug. 22, 2022).

(S8) (a) Jensen, P.; Bunker, P. R. The Potential Surface and Stretching Frequencies of $\tilde{X}\,^3B_1$ Methylene (CH_2) Determined from Experiment Using the Morse Oscillator-Rigid Bender Internal Dynamics Hamiltonian. *J. Chem. Phys.* **1988,** *89,* 1327−1332. (b) Gaspar, P. P.; Hammond, G. S. Spin States in Carbene Chemistry. In *Carbenes*; Moss, R. A., Jones, M., Jr., Eds.; Wiley: New York, 1975; Vol. 2, Chapter 6, pp 207−362.

(S9) According to the (U)CCSD(T)(full)/cc-pVTZ//(U)ωB97X-D/cc-pVTZ + 1.3686(E_{ZPVE}) theoretical model.
